# The reliability characteristics of the REFLECT rubric for assessing reflective capacity through expressive writing assignments: A replication study

**DOI:** 10.1007/s40037-020-00611-2

**Published:** 2020-08-17

**Authors:** Lawrence Grierson, Samantha Winemaker, Alan Taniguchi, Michelle Howard, Denise Marshall, Joyce Zazulak

**Affiliations:** 1 Department of Family Medicine, McMaster University, Hamilton, ON Canada; 2 McMaster Education Research, Innovation, and Theory (MERIT), Faculty of Health Sciences, McMaster University, Hamilton, ON Canada

**Keywords:** Reflection, Assessment, Reliability, Summative, Formative

## Abstract

**Introduction:**

The medical education community has implemented writing exercises that foster critical analysis and nurture reflective capacity. The REFLECT rubric (Wald et al. 2012) was developed to address the challenge of assessing these written reflections. The objective of this replication work is to explore the reproducibility of the reliability characteristics presented by the REFLECT developers.

**Methods:**

Five raters evaluated narratives written by medical students and experienced clinicians using the REFLECT rubric. Reliability across rubric domains was determined via intraclass correlation coefficient and internal consistency was determined via Cronbach’s alpha.

**Results:**

Intraclass coefficients demonstrated poor reliability for ratings across all tool criteria (0.350–0.452) including overall ratings of narratives (0.448). Moreover, the internal consistency between scale items was also poor across all criteria (0.529–0.621).

**Discussion:**

We did not replicate the reliability characteristics presented in the original REFLECT article. We consider these findings with respect to the contextual differences that existed between our study and the Wald and colleagues study, pointing particularly at the possible influence that repetitive testing and refinement of the tool may have had on their reviewers’ shared understanding of its use. We conclude with a discussion about the challenges inherent to reductionist approaches to assessing reflection.

## Introduction

Reflection is a metacognitive activity that involves thinking intentionally about performance before, during, or after situations with the aim of detecting and characterizing the mental models that underpin the decisions and actions relevant to the performance outcome [1, 2]. These models refer to the representations that one has of the relationships that exist between various aspects of the world in which they perform. When healthcare practitioners reflect, it allows them to perceive information from clinical encounters in a way that has the potential to inform their practice in future encounters. As such, it is seen as an essential habit to nurture in new physicians. Accordingly, many medical training programs have adopted writing exercises as a way to develop the capabilities of reflection. In these and other similar assignments, learners write personal stories, or ‘narratives’, of professional encounters as a way to explore their own experiences within those stories. In doing so, learners are encouraged to attend to the emotions, memories, biases, sensory experiences, and social interactions that may have been meaningful within that encounter and to consider the way in which they influenced their ability to communicate the perspectives of patients [3–10], perform critical analyses [11–13], construct clinical meaning [14], understand practitioner roles [15], and appreciate personal values and beliefs [16]. However, the integration of these exercises within the curriculum has presented a difficult question: *what role do written narratives play in helping us determine our residents’ competence as reflective practitioners?*

The literature regarding the use of written narratives in medical education reveals a prominent line of research concerned with the development of tools to facilitate the assessment of reflective writing [5, 6, 12, 13]. Among the most widely published of these is the Reflection Evaluation for Learners’ Enhanced Competencies Tool (REFLECT), which presents a matrix wherein five criterion characteristics of the written narrative are assessed on a 4-level scale that spans from ‘*non-reflective*’ to ‘*critically reflective*’ and that offers a provision for specifying whether any critical level reflection was indicative of confirmatory or transformative learning [5, 12]. The tool also prompts users to justify their rankings for each criterion, which involves generating written commentary pertaining to the aspects of the text that are particularly representative of the chosen ratings. These justifications are intended to serve as the foundation for the delivery of feedback to learners. In this way, the tool apparently serves two purposes. The first is to standardize the assessment of medical trainees’ capability to reflect through examination of their written narratives. The second is to guide faculty reviewers as they structure meaningful feedback. The tool’s exhibited characteristics of good reliability (ICC single measures = 0.632)—albeit with considerable variation in reliability coefficients across developmental iterations (0.376–0.748)—support its suitability for assessing written reflections. However, reliability characteristics are often directly relevant to the contexts in which a measurement tool is developed and tested, and it is therefore important to replicate psychometric examination of such tools before applying them in new educational contexts [17, 18].

In this Replication study, an investigation of the reliability characteristics of the REFLECT rubric in our own context is presented. This involved recruiting five medical educators to read and assess the reflective writing of a group of medical students and a group of experienced family physicians by way of the final version of the REFLECT tool described by Wald and colleagues [12]. These ratings provided the data foundations for appraisal of whether the rubric’s reliability characteristics were reproducible in our context.

## Methods

### Participants

Five (5) faculty educators from the medical education community at McMaster University (Hamilton, Canada) were recruited as raters for this study. Care was taken to recruit individuals with more than 5 years of experience at delivering writing curricula for the purposes of promoting reflection. All raters provided informed consent according to the guidelines set out by the Hamilton Integrated Research Ethics Board (HIREB) and the Declaration of Helsinki (2013) before participating in this study.

### Data collection

Two sets of writing assignments were acquired. One set was written by 15 first-year medical students during their first term at McMaster University. The second set of submissions were written by 15 clinician faculty from the Department of Family Medicine at McMaster University. Both groups had written pieces in response to a prompt that was selected from McMaster’s undergraduate professional competency curriculum on the basis of its relevance to both groups:

*During this time in your life, self-care is particularly important. How are you caring for your whole being—body, mind and spirit—during this time of your life? Are there particular strategies/ideas for achieving balance that you might share with your colleagues?*

All writers also provided informed consent according to the guidelines set out by the HIREB before their narratives were included in this study.

### The tool

The REFLECT rubric comprises five essential criteria that are rated on one of four levels. The five essential criteria are the spectrum of written exploration, the writer’s presence in the written work, the quality of description of the concerning issue, the writer’s attention to their own and others’ emotions, and the overall meaning the writer derives from the explored experience. For each written narrative, each of the five criteria as well as the overall written work is rated as either non-reflective, introspective, reflective, or critically reflective [12]. An assessment of the reflection associated with the overall written work was included because Wald and colleagues indicated including such a rating in their development process for research purposes. We recognize that students do not typically receive this information as part of assessments involving this tool. Raters are required to provide written justifications for each reflective level assigned to each criterion.

### Protocol

All five raters attended a 2-hour introductory workshop during which they were introduced to the assessment tool. As a training exercise during this session, each rater used the rubric independently to assess a set of two additional written submissions provided by consenting family medicine residents. The raters then participated in a facilitated discussion wherein they reviewed their ratings and arrived at a common understanding and approach to using the tool.

Following this orientation, each rater was assigned written submissions from the study sample pseudo-randomly. The pseudo-randomization process ensured that each submission was reviewed by two different raters, and that no two raters assessed the exact same submissions. All rubric assessments were completed by hand with pen-and-paper and returned within three weeks to the research team.

### Analysis

The four levels of reflection ability were numbered one (*non-reflective*) through four (*critically reflective*). In order to assess inter-rater reliability, we applied the statistical methods of Wald and colleagues (2012), and determined the single measures intraclass correlations associated with the ratings of each of the four criteria as well as the overall written work [19]. We also used Cronbach’s alpha to report the internal consistency of the ratings. Intraclass correlation values less than 0.5 are indicative of poor reliability, values between 0.5 and 0.75 indicate moderate reliability, values between 0.75 and 0.9 indicate good reliability, and values greater than 0.90 indicate excellent reliability [20].

## Results

The full spectrum of the REFLECT scale was used by raters. Tab. 1 shows the scoring of the submissions for each rater.

**Table 1:** Distribution of participant ratings across scale levels and subdomains

	First rater (%)	Second rater (%)
*Writing spectrum*		
– Habitual action (non-reflective)	25.4	22.0
– Thoughtful action or introspection	15.3	11.9
– Reflection	33.9	42.4
– Critical reflection	25.4	23.7
*Presence*		
– Habitual action (non-reflective)	18.6	13.6
– Thoughtful action or introspection	27.1	35.6
– Reflection	27.1	20.3
– Critical reflection	27.1	30.5
*Description of disorienting dilemma*		
– Habitual action (non-reflective)	20.7	10.7
– Thoughtful action or introspection	22.4	35.7
– Reflection	34.5	35.7
– Critical reflection	22.4	17.9
*Attending to emotions*		
– Habitual action (non-reflective)	24.1	24.1
– Thoughtful action or introspection	29.3	27.6
– Reflection	13.8	27.6
– Critical reflection	32.8	20.7
*Analysis and meaning making*		
– Habitual action (non-reflective)	20.3	15.3
– Thoughtful action or introspection	22.0	27.1
– Reflection	30.5	32.2
– Critical reflection	27.1	25.4
*Overall rating*		
– Habitual action (non-reflective)	28.3	24.1
– Thoughtful action or introspection	17.0	17.2
– Reflection	26.4	34.5
– Critical reflection	28.3	24.1

Tab. 2 shows the single measures intraclass coefficient and Cronbach alpha statistics for each criterion and overall rating. Intraclass coefficients demonstrated poor reliability for ratings of all criteria. Moreover, the internal consistency between scale items was poor across all criteria.

**Table 2:** Reliability characteristics of participant ratings

	ICC single measures (95% CI)	Cronbach alpha
Writing spectrum	0.368 (0.125–0.570)	0.532
Presence	0.367 (0.125–0.567)	0.529
Description of disorienting dilemma	0.452 (0.220–0.635)	0.621
Attending to emotions	0.350 (0.107–0.555)	0.514
Analysis and meaning making	0.384 (0.145–0.581)	0.549
Overall rating	0.448 (0.222–0.628)	0.613

## Discussion

We sought to determine whether ratings of the reflective quality of written narratives generated through application of the REFLECT rubric in our own context would yield reliability characteristics similar to those described by Wald and colleagues in their published presentation of the tool and its development [12]. Of note, our analysis revealed reliability characteristics that were poor and considerably lower. Where Wald and colleagues (2012) achieved intraclass coefficients as high as 0.748 for overall reliability [12], we realized a coefficient for similar ratings of only 0.448, with coefficients associated with the tool’s various components registering mostly at lower levels than that.

It is important to keep in mind that characteristics of good reliability are often directly relevant to the contexts in which a tool is tested [17, 18], and, in this regard, we can acknowledge a number of differences between the context of our application and that of Wald and colleagues. For instance, Wald and colleagues tested the REFLECT tool exclusively on narratives written by undergraduate medical students, whereas our narratives were authored by a mix of medical students and clinical faculty. Moreover, each bit of reflective writing in our study was rated by two reviewers, while the tool developers typically employed three raters in their tests; a potential limitation of our replication. However, from our view, perhaps the most salient contextual difference is that the rubric presented by Wald and colleagues was developed over a series of three iterations and five pilot tests, all conducted within a year (2009–2010), and largely at their own institution (Brown University, Providence, RI, USA), while our use of the tool involved a single, later application at a different institution than the one at which it was developed (McMaster University, Hamilton, Canada). In this regard, we speculate that Wald and colleagues’ iterative process of development may not only have improved the technical components of the tool, but also their collective ability to apply it towards the reflection construct. That is, their raters, through the repetitive use, discussion, and refinement described in the 2012 paper, may have constructed, amongst themselves, a shared understanding of the rubric’s criteria and, in turn, an approach to using the tool that improved its overall reliability. The notion here is that the good reliability statistics do not emerge solely as a function of the tool, but through an intersection between the assessors’ application of the tool and their understanding of what it is designed to measure. This sort of consensus understanding building may have occurred because the same raters were included across testing iterations or by way of a progressively-refined set of directions provided to raters by the research team; although, we recognize neither is discernible from the Wald report. While we attempted to prepare our assessors through a pre-study rater training process, this may not have been sufficient to reconcile any fundamental differences in the way our raters and their raters understood the tool’s constructs. Indeed, our training may have amplified differences.

Importantly, this exercise has strengthened our belief that measuring reflection through written narrative is potentially flawed, running counter to the philosophical underpinnings of reflection [21–23]. Where most theories of reflection endorse imaginative exploration of cognitive, affective, physical, and verbal experiences when making meaning of vague and uncertain circumstances [24, 25], the creation of a tool that simplifies reflection into discrete components limits learners ability to be expansive and promotes their tendency to cater their writing to the goal of ‘scoring well’ [26, 27]. From our perspective, a written reflection serves as a catalyst for formative dialogue between learners and instructors [28], much like that which occurs during simulation debriefing [2, 14, 29, 30]. The idea is that the writing exercise prompts learners to think upon a recent experience and to construct an account of how aspects of that experience influenced their decisions and actions. In doing so, learners are challenged to identify influences and interactions that were not previously noticed, and to use these revelations to formulate strategies that can be brought forward into future experiences. However, learners (at any stage of expertise) are typically not capable of identifying all the relevant aspects that impart influence on a clinical encounter on their own [30, 31]. Through writing, however, they can share their accounts with an instructor; and by reading, these instructors can come to a deeper appreciation of the representations and assumptions that underpin learner behaviour. In doing so, bespoke feedback that targets learners’ needs can be generated. In this way, the writing supports formative assessment, but does not, in and of itself, constitute the object of assessment.

The REFLECT developers hold that the tool supports this formative process; yet its reduction of the reflection to a set of pre-defined criteria highlights a fundamental challenge in incorporating reflection activities into medical education curricula. Recent shifts in medical training have given way to an education paradigm that increasingly distills medical practice into defined professional activities, each of which that can be directly observed. In this regard, we can understand the application of a reductionist perspective onto the construct. Simply put, by presenting reflection as a set of discrete components, the educator is provided a means of assessment for this important physician activity that fits into the competency-based model. However, through reductionist assessment, the educator also runs the risk of assuming that the degree of competence that a learner has for reflection can be determined through the critical reading of his or her written account. Accordingly, the narrative may be characterized as something of a final report of the whole reflection process, and the assessment may not account for any reflection that occurs as a consequence of the writing.

## Conclusion

Through this replication exercise, we were afforded the opportunity to consider more fulsomely our understanding of the factors that come to bear when assessing reflection in medical education. In doing so, we have highlighted that it is vitally important for the medical education community to come to a shared understanding of how reflection is conceptualized and utilized within the curriculum. In particular, we echo the recommendations of De la Croix and Veen (2018), and advocate for approaches to reflection that abandon the checklist and encourage learners to reflect freely, employing styles that nurture and protect the deeply personal nature of self-exploration [24].
